# TEMPO-Oxidized Cellulose Nanofibers: A Potential Bio-Based Superabsorbent for Diaper Production

**DOI:** 10.3390/nano9091271

**Published:** 2019-09-06

**Authors:** Josefina Patiño-Masó, Ferran Serra-Parareda, Quim Tarrés, Pere Mutjé, F. Xavier Espinach, Marc Delgado-Aguilar

**Affiliations:** 1 IRQV Institute, Department of Nursing, University of Girona, Emili Grahit, 77-17003 Girona, Spain; josefina.patino@udg.edu; 2 LEPAMAP Research Group, University of Girona, Maria Aurèlia Capmany, 61-17003 Girona, Spain; ferran.serra@lepamap.udg.edu (F.S.-P.); joaquimagusti.tarres@udg.edu (Q.T.); pere.mutje@udg.edu (P.M.); 3 PRODIS Research Group, University of Girona, Maria Aurèlia Capmany, 61-17003 Girona, Spain; francisco.espinach@udg.edu

**Keywords:** cellulose nanofibers, aerogels, superabsorbent, absorption properties, diapers, nanocellulose

## Abstract

Single-use plastics are expected to disappear, mainly due to the rise of stricter regulations to combat their impact on the environment. As an example, the recent European Directive on Single-Use-Plastics (SUP) will be implemented between 2021 and 2024 and will directly prohibit the use of some SUP. Baby diapers are one of the most used single-used products in our daily lives, and it is estimated that most of the ~4000 diapers that each baby uses in their life go to landfill. Such diapers usually contain superabsorbent polymers (SAP) that are based on acrylic acid-acrylamide mixtures with high water retention capacity, but they are neither bio-based nor biodegradable. In this work, we have developed bio-based superabsorbent aerogels made of cellulose nanofibers (CNF) and propose their potential use in baby diapers. TEMPO-oxidized CNF at different oxidation degrees were prepared and tested. The obtained CNF exhibited higher free swelling capacity (FSC) than the commercial fluff pulp (ranging from 117.62% to 245.21% higher) and also than the diaper absorbent, except for CNF-5 (ranging from 31.56% to 54.55%), even under compression. Overall, the present work shows a case study where CNF could have a potential application with market opportunities.

## 1. Introduction

In September 2015, the Heads of State and Government and High Representatives reached a historic decision on a comprehensive, far-reaching, and people-centered set of universal and transformative Goals and Targets. Thus, they established the 2030 Agenda for Sustainable Development, which contained 17 Sustainable Development Goals (SDG). It is intended that these goals stimulate different areas of importance for society and the planet: the people, the planet, prosperity, peace, and partnership [1]. Among the different 17 SDG, goals 13, 14, and 15 focus on combating climate change, on sustainable growth, and on the preservation of natural resources.

More recently, in May 2019, the European Parliament agreed to implement a Directive proposed by the European Commission to tackle marine litter by 2021–2024 [2]. Among the different strategies and measures of the proposed Directive, there will be a ban on selected single-use products made of plastic, measures to reduce their consumption, and producers will be held to higher responsibilities [3].

However, the abovementioned Directive does not list all the products that contain single-use plastics such as baby diapers, or products for adult incontinence, agricultural application, and feminine care. Even though most of these products are based on wood-derived materials, mainly fluff pulp, those that are absorbent contain superabsorbent polymers (SAP). SAP are cross-linked, three-dimensionally structured hydrogels that are able to absorb significant amounts of water [4,5]. These SAP are usually based on acrylic acid-acrylamide polymer mixtures and their landfilling may cause a significant environmental impact since they are neither bio-based nor biodegradable [6]. In fact, in 2007, The Nappy Alliance informed the Parliament of the United Kingdom that disposable diapers accounted for around 4% of household waste and that such products take approximately 500 years to decompose in the case of landfilling [7]. They also estimated that a baby uses about 4000 diapers on average (about 120 kg) and most of them go to landfill, which represents about 8 million diapers per day in the UK [8]. Hence, although there are already natural-based SAPs from chitosan, starch, carrageenan and starch, there is no doubt that finding a greener solution is an urgent matter that needs to be tackled quickly [9,10]. The state of Vanuatu for example, a small archipelago in the Pacific Ocean with a population of about 0.3 million people distributed across 65 islands, has already decided to ban disposable diapers [11].

Cellulose is the most abundant biopolymer on Earth. It is present in plants and trees, and it is widely used for paper and paperboard production [12]. Nonetheless, cellulose-based materials are also used in other products and applications such as pharmaceuticals, rheological modifiers in paints and cosmetics, food additives, and absorbents [13,14,15].

In recent years, the use of different cellulosic pulps as raw materials for the production of cellulose nanofibers (CNF) has gained significant interest among the scientific and technological community [16,17,18]. CNF can be defined as cellulosic fibers, containing both amorphous and crystalline regions, with diameters in the nanoscale and lengths of a few micrometers. Usually, the production process of CNF consists of two steps: pretreatment and fibrillation. Pretreatment can be chemical-based or enzymatic. This is usually conducted to ease the fibrillation step, since it avoids clogging in high-pressure homogenizers or because it softens the fibers prior to grinding, depending on the selected fibrillation method. In addition, different pretreatments will give rise to CNF with significantly different characteristics that can be used in a wide range of applications [19]. Among these pretreatments, TEMPO-mediated oxidation [20], enzymatic hydrolysis [21], mechanical refining [22], acid hydrolysis [23], and carboxymethylation [24] are worthy of mention. Thus, different CNF morphology, mechanical and optical properties, as well as production costs will be obtained depending on the production method [19,25].

The main features of CNF are their high specific surface, excellent mechanical properties, and their ability to be chemically modified for several purposes. Also, a wide variety of CNF-based products can be produced including aerogels, nanopapers, and hydrogels [15,26,27,28,29,30]. In addition, CNF have potential applications in diverse sectors such as biomedicine, environmental science, paper and board production, electronics, and plastic composites, among others [14,31,32,33,34]. Although this versatility offers CNF great opportunities in several fields, their renewable character is one of the main drivers of their success [35].

From the abovementioned CNF-based products, aerogels are gaining interest due to their huge specific surface, interesting mechanical properties, low weight and the wide range of applications they can be used for. CNF-based aerogels have been used as selective absorbents of water-oil mixtures, as adsorbents of heavy metals and dyes, as supercapacitors, and even as thermal and sound insulators [36,37,38,39,40,41].

CNF have been extensively investigated as potential absorbents for several applications. In most of the cases, CNF have been processed to obtain partially modified aerogels and sponges that can be used as selective absorbents for oil and water mixtures [28,39,40,42,43]. However, cellulose nanofibers have also been investigated as potential SAP substitutes, mainly due to the presence of COO^−^ groups that can create hydrogen bonding with water molecules [44]. In addition to this, the fibril morphology also imparts a significant effect on water absorption capacity of cellulose materials. In fact, a recent study suggests that the absorption properties of fibers and CNF should be reported as a key characterization parameter [45]. The paper-making industry for example, already uses mechanical refining in order to improve the swelling capacity of fibers. Thus, it is well known that the absorption properties of fibers can be significantly improved by enhancing their specific surface, which has also been studied in nanocelluloses [12,19,46]. Chatterjee and Makoui investigated the water absorption capacity of microfibrillated cellulose (MFC) obtained by means of mechanical methods in a 1% saline solution, obtaining a retention of 10 g of water per gram of cellulose, value that was even increased to 20 g/g for other MFC materials [47,48]. In a more recent work, Brodin and Theliander found that if cellulosic fibers were pretreated by means of TEMPO-mediated oxidation, the free swelling capacity (FSC) could be increased up to 60 g/g after 30 min in contact with a saline solution. In this work, the amount of NaClO during the oxidation was limited to 4.2 mmol/g of pulp, leading to a maximum oxidation degree (carboxylate groups) of 1.64 mmol/g [49]. In a similar context, Mendoza et al. found that the absorption capacity of nanocellulose-based aerogels and foams can be manipulated depending on the processing conditions, apart from the oxidation degree [50]. Thus, the authors found that at moderate carboxylate concentration (1.2 mmol/g) it is possible to absorb 60 g/g of saline solution, while it can be increased up to 120 g/g when deionized water is used as sorbent.

For all the above, in this work we aimed to develop superabsorbent products based on CNF aerogels for their application as diapers absorbents in order to substitute the current SAP by bio-based and biodegradable products.

## 2. Materials and Methods

### 2.1. Materials

Commercial dried bleached kraft eucalyptus pulp (BKEP) was kindly provided by ENCE–Celulosas y Energía, S.A. (Navia, Spain) and was used as the raw material for the production of cellulose nanofibers (CNF). Fluff pulp was kindly provided by Stora Enso (Nymölla, Sweden,) and commercial diapers were purchased in a local supermarket. All the reagents used for the TEMPO-mediated oxidation, ionic cross-linking, CNF characterization, and the preparation of the saline solution were supplied by Sigma–Aldrich (Saint Louis, MO, USA). Distilled water was used in all suspensions and solutions.

### 2.2. Preparation of Cellulose Nanofibers

CNF were prepared by TEMPO-mediated oxidation at basic pH, according to a previously reported methodology, but with a lower amount of TEMPO catalyst (from 16 to 2 g/kg) [20,25]. This reduction on the amount of catalyst allows us to lower costs while preserving the properties of CNF, as confirmed in a previous study [25]. In a typical experiment, 3 g of NaBr and 0.06 g of TEMPO were suspended and dissolved in water for 10 min under gentle stirring. Then, 30 g of fibers (BKEP) were incorporated into the solution and kept under stirring for 10 min to assure a good dispersion of all substances. After this, a 14–15 vol.% NaClO solution was added dropwise onto the slurry. The amount of NaClO was varied in different batches in order to obtain different oxidizer amounts, ranging from 5 to 25 mmols/g of fiber. In all cases, 0.5 M NaOH was added after the oxidizer until the pH stabilized at a value of 10. The reaction was stopped by filtering and washing the oxidized fibers with distilled water several times.

**Figure 1:**
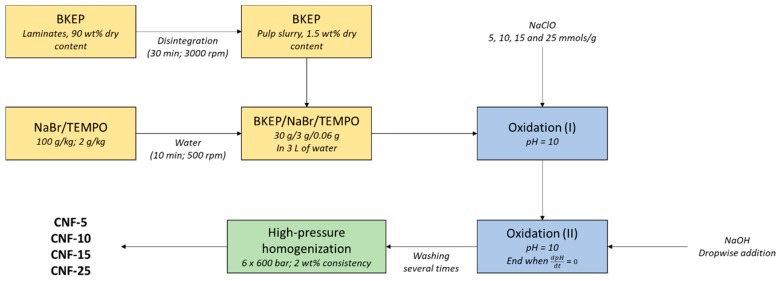
Experimental diagram of the production of TEMPO-oxidized cellulose nanofibers (CNF).

After washing the fibers, they were suspended in water to a consistency of 2 wt%. Then, the fibers were passed through a high-pressure homogenizer (PANDA PLUS 2000, GEA Niro Soavi, Parma, Italy) operating at 600 bar. The process was repeated six times and the resulting suspension was kept at 4 °C until further characterization and use. Depending on the oxidizer amount, the different samples were named as CNF-5, -10, -15 and -25. The whole process is summarized in Figure 1.

In all the processes involved in the production of CNF, energy consumption was continuously measured with a Circutor CVM-C10 (three-phase lines) (Barcelona, Spain) and a Socomec Diris A20 (single-phase lines) (Barcelona, Spain). The production costs of the obtained CNF were calculated by assuming a cost of energy of 0.08 €/kWh and by factoring in the purchasing cost of each reagent. Equipment depreciation and personnel costs were not considered for this estimation.

### 2.3. Characterizatoin of Cellulose Nanofibers

Prior to homogenization, the carboxyl content (CC) of the oxidized fibers was determined by conductimetric titration. In a typical experiment, 100 mg of fibers were suspended in 15 mL of 0.01 M of HCl and kept under mixing for 10 min in order to exchange the Na^+^ cations from the COO^−^ group for H^+^. Then, the suspensions were titrated by adding 0.1 mL increments of a 0.01 M NaOH solution while recording the conductivity of the suspensions. The curve exhibited the presence of a strong acid (excess of HCl) and a weak acid (carboxylic acid). Hence, the carboxyl content is given by the following Equation:

CC = 162 × (V_2_ − V_1_) × c × [(w − 36 × (V_2_ − V_1_)] (1)

where, V_1_ and V_2_ are the equivalent volumes of the added NaOH in liters, c is the concentration of the NaOH (0.01 M), and w is the dry weight of the sample in grams.

The cationic demand (CD) was determined using a particle charge detector Mütek PCD04 (BTG Instruments, Weßling, Germany). First of all, 0.04 g of CNF were diluted in 1 L of water and dispersed in a pulp disintegrator for 10 min at 3000 rpm. From the suspension, 10 mL were taken and mixed with 25 mL of 0.01 N polyDADMAC (Polydiallyldimethylammonium chloride) for 5 min under gentle stirring. Next, the suspension was centrifuged for 90 min at 4000 rpm in order to obtain two phases. Finally, 10 mL of the supernatant was collected and titrated with 0.01 N Pes-Na (poly(ethylene sulfonate) sodium salt). The CD was calculated as follows:

CD = (C_poly-D_ × V_poly-D_) − (C_Pes-Na_ × V_Pes-Na_)/W_sample_ (2)

where, C_poly-D_ and V_poly-D_ are the concentration and volume of the polyDADMAC respectively, C_Pes-Na_ and V_Pes-Na_ are the concentration and volume of Pes-Na, and W_sample_ is the dry weight of the sample. As reported elsewhere, it is possible to calculate the specific surface and diameter of CNF with the values of CD and CC (in µeq-g/g) based on some assumptions related to the geometry of CNF and the adsorption mechanism of polyDADMAC [22,25]. Both parameters were also calculated.

The yield of fibrillation, expressed as the relative amount of fibers that have been fibrillated and are in the nano domain, was also determined. For this, we centrifuged a 0.2 wt% CNF suspension at 4500 rpm for 20 min. While the nanometric fraction remained in the supernatant, those fibers that were not fibrillated precipitated to the bottom of the container. Then, the supernatant was discarded, and the solids were oven-dried until constant weight. The yield of fibrillation was then calculated according to the following Equation:

Yield = [(1 − Ws)/Wi] × 100(3)

where, Ws is the dry weight of the sediment and Wi is the initial dry weight of CNF.

The transmittance of the diluted CNF (0.1 wt%) was measured using a UV-Vis Shimadzu spectrophotometer UV-160A, set in the range between 800 and 400 nm. Distilled water was used as a reference.

The degree of polymerization (DP) was determined from intrinsic viscosity measurements, adapting the UNE 57-039-92 standard for CNF and using cupriethylenediamine as a solvent. The average molecular weight was calculated using the equation proposed by Mark-Howink-Sakurada:

η = K × M^a^, (4)

where, η is the intrinsic viscosity (dL/g), a and K are constants (0.76 and 2.28, respectively) that depend on the polymer-solvent system, and M is the molecular weight, as reported by Henriksson et al. [21].

Finally, the water retention value (WRV) of CNF was measured as follows. CNF solutions were centrifuged in bottles equipped with a nitrocellulose membrane (0.22 µm of pore size), which separates the non-bonded water out of the CNF solution. After centrifugation (2400 rpm for 30 min), the wet cake was collected, weighted, and dried at 105 °C until constant weight. The WRV was calculated according to the following equation:

WRV = (Ww − Wd)/Wd,(5)

where, Ww is the wet weight of the cake and Wd is the dried weight of the cake.

### 2.4. Preparation of the Aerogels

First, the obtained CNF were diluted to 0.5 wt%. The suspensions were then poured into a metaling dish and frozen at −80 °C for 24 h. Next, the frozen samples were freeze-dried in a lyophilizer until constant weight was obtained, thus assuring that all the water was removed. Samples were stored at room temperature until further use and characterization.

### 2.5. Characterization of Aerogels and Commercial Diapers

Samples were characterized in terms of morphology (field emission scanning electron microscopy, FE-SEM), free swelling capacity (FSC) and centrifuge retention capacity (CRC). In the case of aerogels, compression tests were performed both at 50% and 100% deformation and the loss of water was quantified. Only in the case of 50% deformation, the compression strength was recorded.

FE-SEM of the aerogels was conducted using a Zeiss DSM 960 microscope. Samples were bound to the metal holder using carbon tape and coated with a thin layer of gold.

FSC and CRC were determined by soaking the different samples in a saline solution (0.9 wt% NaCl). The absorption tests were based on the European Disposable and Nonwovens Association (EDANA) standard methods. EDANA is the trade association representing most manufacturers of baby diapers in Europe, Middle East and Africa (EMEA). However, both standards were slightly modified to make them suitable for CNF aerogels. These tests were also performed on the absorbent of the commercial diapers. To facilitate comprehension, we expressed the results as gH_2_O/g, even though saline solution was used.

For FSC calculation, the different samples were soaked in the saline solution for different times, ranging from 5 to 60 min. The weight was recorded after every soaking and compared to the initial dry weight of sample. This was performed in order to obtain the water absorption curves as a function of time, and to obtain the saturation point of the samples.

For CRC calculation, we used the same methodology used in WRV calculation, where the aim was to retain all the CNF in the wet cake of the centrifuge bottles. For this test we used the FSC samples that were soaked for 60 min.

Compression tests were performed in an Instron universal testing machine harboring a 250 N load cell using a cross-head velocity of 2 mm/min. For this test we used wet aerogels (60 min soaked FSC samples) with a cylindrical shape, and an average diameter of 40 mm and a height of 50 mm.

## 3. Results and Discussion

As explained above, we characterized CNF obtained with different oxidizer amounts during TEMPO-mediated oxidation according to previously reported methods. The results are shown in Table 1.

The results show that higher values of transmittance, yield of fibrillation, and WRV were achieved with increasing amounts of oxidizer (NaClO), while the values of DP were reduced. The yield of fibrillation achieved an almost 100% in the case of CNF-25 and all yields are in accordance with those reported in the literature [29]. Taking into account that the fibrillation strategy was the same in all cases, the differences on the properties can be directly attributed to the different treatment conditions during TEMPO-mediated oxidation. It is known that the main mechanism behind this treatment is the selective oxidation of C6 primary hydroxyls of cellulose to C6 carboxylate groups and, more precisely, to COONa groups when the oxidation is carried out at basic pH [20,51]. Such carboxyl groups have greater volume than hydroxyl groups even in their acidic form. In addition, they enable greater electrostatic interactions between fibers, which promotes fibrillation during high-pressure homogenization. Moreover, it has been reported that this treatment also introduces aldehyde groups (CHO) [52].

The higher transmittance exhibited by CNF suspensions obtained with higher amounts of oxidizer may be correlated with the higher yield of fibrillation, but also with a lower size of CNF, since the size of CNF has a direct impact on light scattering [53].

As expected, the DP of CNF decreased as the amount of NaClO increased. This was previously observed by Tarrés et al., where it was found that TEMPO-mediated oxidation fibrillates fibers. This results in the separation of fiber bundles which leads to thinner fibers, and in the breakage of cellulose chains which in turn decreases fiber length [29]. In fact, Shinoda et al. [54] reported that there is a relationship between the length of the TEMPO-oxidized cellulose nanofibers and their DP. This relationship is the following:

Length (nm) = 4.286 × DP − 757,(6)

While we have used this relationship in previous works [17,52], it did not seem to work in this case since we obtained a length of approximately 1 nm when we introduced the data of CNF-25 into this relationship. This result is impossible because such CNF would have greater diameter than length. In fact, Shinoda et al. already found that fibrils exhibiting DP below 200 were out of the range of this equation. Nonetheless, there is no doubt that TEMPO-mediated oxidation decreases the molecular weight of cellulose, leading to shorter chains and fibers.

WRV is a measure of how much water is chemically bonded to fibers. As seen in Table 1, WRV increased from 8.21 to 13.08 g of water per g of CNF upon increasing amounts of NaClO. We hypothesized that this is due to an increase in the specific surface of CNF, which is later demonstrated in the paper.

**Figure 2:**
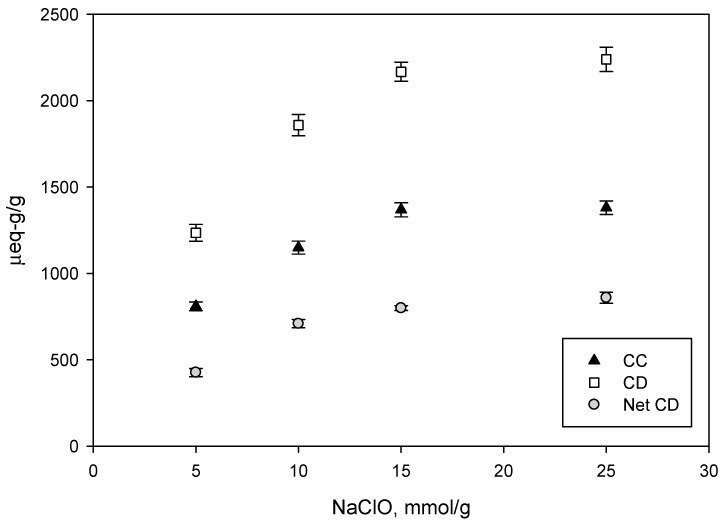
Evolution of the carboxyl content (CC) (black), cationic demand (CD) (white) and Net CD (grey) with increasing amounts of NaClO.

Figure 2 shows the evolution of cationic demand (CD), carboxyl content (CC) and the net cationic demand (Net CD). As explained above, TEMPO-mediated oxidation is based on the oxidation of the C6 hydroxyl group to a carboxyl group. Thus, we expected an increase in the amount of carboxyl groups with higher amounts of oxidizer. However, the CC and CD remained almost constant for NaClO values between 15 and 25 mmol/g and we believe this is due to the saturation of the cellulose surface by carboxyl groups. Since the CC and CD of CNF-15 and CNF-25 are very similar, as are the properties shown in Table 1, the use of CNF-25 for certain applications does not seem to be justified.

As a comparison, Lu et al. prepared TEMPO-oxidized CNF from bleached bagasse pulp with 4, 6, and 8 mmols/g of NaClO. The obtained CC values were 730, 1080, and 1290 µeq-g/g respectively, which are slightly higher values than the ones obtained in the present work for a similar amount of NaClO [26]. On the other hand, Saito and Isogai prepared TEMPO-oxidized CNF with 2.42 mmols/g of NaClO and obtained a CC of 480 µeq-g/g, which matches very well with our work.

The CD increased with higher oxidizer amount and higher anionic character of CNF, as expected (Figure 2).

**Figure 3:**
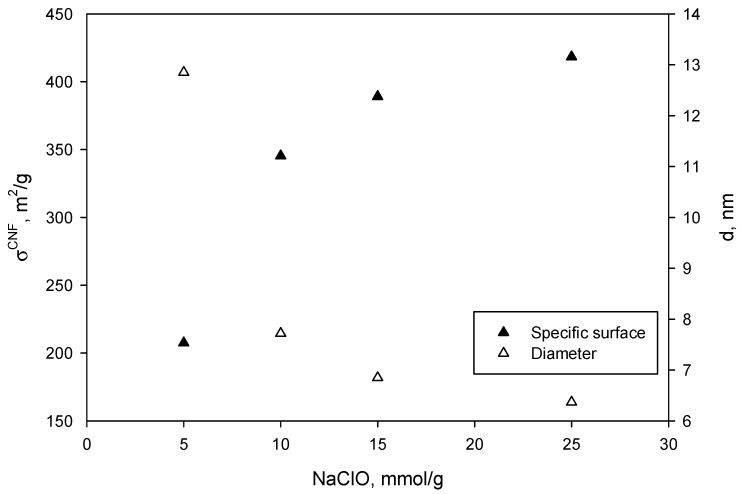
Evolution of the specific surface (black, left vertical axis) and the fiber diameter (white, right vertical axis) with increasing amounts of NaClO.

Net CD can be described as the CD generated due to the increase in the specific surface and it is calculated by subtracting CC from CD (Figure 2). Net CD deserves special mention because it reflects the anionicity of the suspension neglecting the electro-negative charges of carboxyl groups and is thus proportional to the specific surface of the CNF. Hence, this difference corresponds to the amount of cationic polymer unabsorbed by the carboxyl groups. In fact, there are two different types of interaction between polyDADMAC and CNF. On the one hand, there are ionic interactions between the cationic polymer and the carboxylic groups on the cellulose surface. On the other hand, there are surface interactions due to Van der Waals forces [55,56]. If both mechanisms are assumed to occur simultaneously and the polyDADMAC forms a monolayer, the specific surface of CNF can be obtained by calculating the specific surface of a single molecule of polyDADMAC. This can be performed because of the high molecular weight of the polyDADMAC used during CD determination (107 kDa). However, we previously found that excessively high molecular weight polyDADMAC, unlike low molecular weight polymers, was not completely adsorbed onto the CNF surface due to conformational and steric constrains [32]. In addition to this, the degree of polymerization of the polyDADMAC was 662. With all this data, the specific surface of a mol of polyDADMAC was calculated using the following Equation:

(7) $$\sigma^{\mathrm{DADMAC}}=662\times N\times \pi \times d\times l,$$

where, $\sigma^{\mathrm{DADMAC}}$ is the mol’s area, d and l are the calculated diameter and length (0.528 and 0.488 nm, respectively), and N is the Avogadro constant. Thus, the specific surface of a mol of polyDADMAC accounted for 3224 × 10^23^ nm^2^. Then, considering that in one mol of polyDADMAC there are 662 × 10^6^ µeq-g/mol, the specific surface can be also expressed as 4.87 × 10^17^ nm^2^/µeq-g. Next, according to the stoichiometry between hydroxyl, carboxyl, and polyDADMAC, the specific surface of CNF can be calculated as:

(8) $$\sigma^{\mathrm{CNF}}=\left(\mathrm{Net}\;\mathrm{CD}\right)\;\times {\;\sigma }^{\mathrm{DADMAC}},$$

where, $\sigma^{\mathrm{CNF}}$ is the specific surface of one gram of CNF. Finally, assuming a cylindrical geometry for CNF, their average diameter (d^CNF^) can be calculated as follows:

(9) $$d^{\mathrm{CNF}}=\frac{4}{\sigma^{\mathrm{CNF}}\;\times {\;\rho }^{\mathrm{cell}}},$$

where, $\rho^{\mathrm{cell}}$ is the cellulose density (1.6 g/cm^3^). More details about this methodology were reported in previous works [22,25]. The results are shown in Figure 3.

**Figure 4:**
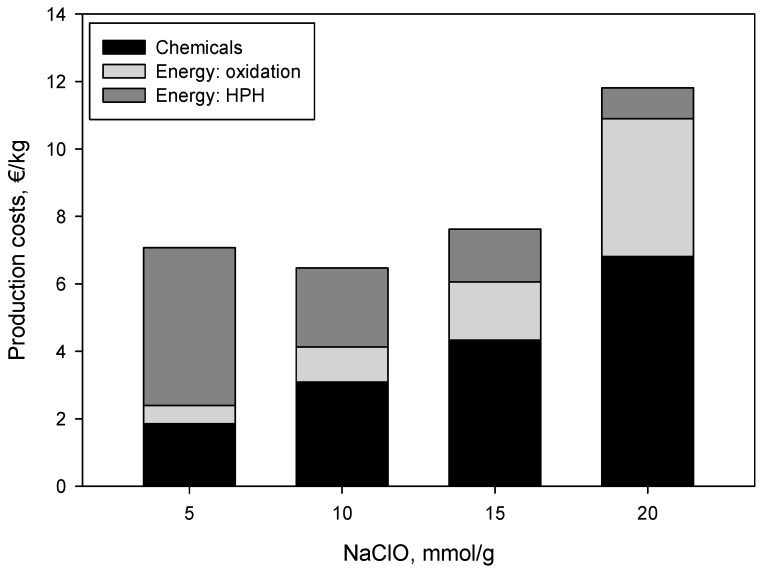
Production costs of the CNF obtained with different amounts of NaClO.

Figure 3 shows the evolution of the specific surface (black, left vertical axis) and the fiber diameter (white, right vertical axis) as a function of NaClO amount. Greater specific surface was achieved with higher amounts of NaClO (similarly to CC, CD, and Net CD) while fiber diameter decreased. The obtained specific surface and diameters are in accordance with previous results for this kind of CNF [17]. In principle, as reported for nanopapers, the higher the specific surface, the more inter-CNF bonds can be created [17]. On the other hand, the present works aims at developing aerogels. When CNF are suspended in water, they generate repulsive forces that can enhance the porosity and the specific surface of the resulting aerogels. In any case, one can expect that as the specific surface increases, the absorption capacity should also be greater [28,39,40,57]. In fact, Figure 4 shows that under the same fibrillation conditions, one can control CNF diameter and their specific surface by adjusting the amount of NaClO.

Taking into account the cost of the different chemicals involved in the TEMPO-mediated oxidation, the energy consumption during this pretreatment, and the energy required for the fibrillation during the high-pressure homogenization (HPH) stage, one can see that the production costs of CNF-25 are much higher than those of CNF-15. In fact, the cheapest CNF were those oxidized with 10 mmol/g of NaClO. The obtained production costs are in accordance with those reported in the literature where prices of industrial orders were taken into account for chemicals (Figure 4) [25].

**Figure 5:**
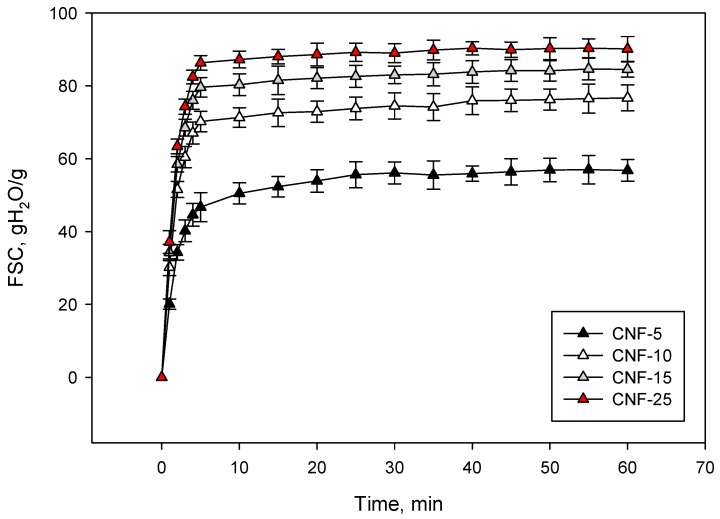
Evolution of free swelling capacity (FSC) as a function of immersion time (in minutes).

As detailed in the previous section, we immersed aerogels in 0.9% NaCl solution for 60 min and recorded the amount of water they retained at different times. As shown in Figure 5, all aerogels reached a saturation point after 5 min of immersion except the one oxidized with 5 mmol of NaClO per g of fiber, for which about 25 min were required for full saturation. This suggests that water penetration into the porous aerogel structure is significantly affected by the specific surface of the obtained nanofibers, probably due to steric effects. In addition, we found that the higher the amount of NaClO used, the more water the aerogels were able to retain. This was in accordance with the WRV results shown in Table 1.

**Figure 6:**
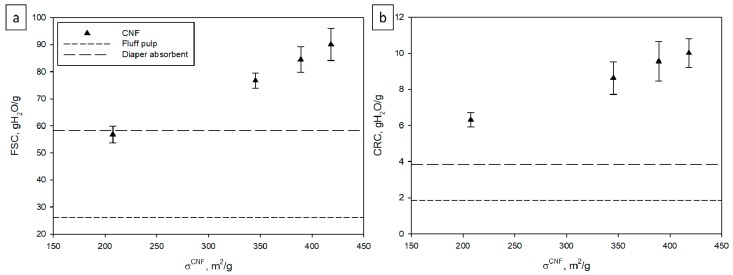
Effect of the specific surface on FSC at 60 min (**a**) and CRC (**b**) of CNF compared to fluff pulp and a commercial diaper absorbent.

We then compared the FSC value at 60 min of each aerogel to those obtained using the commercial fluff pulp and the diaper absorbent upon prior removal from the diaper (Figure 6, left graph). The minimum FSC value of CNF was obtained at a specific surface of about 200 m^2^/g at 56.8 g of water per g of CNF, which was very similar to that obtained with the commercial absorbent but significantly higher than that obtained with the fluff pulp. In relative terms, the FSC values of the CNF-10, CNF-15, and CNF-25 were 31.56, 44.94, and 54.55% higher than the diaper absorbent, respectively. Compared to the commercial fluff pulp, the CNF-5, CNF-10, CNF-15, and CNF-25 exhibited a 117.62%, 193.87%, 223.75%, and 245.21% higher absorption, respectively. We note that the WRV values of the CNF (see Table 1) already pointed to their high-water absorption and retention capacity. As mentioned in the introduction, the absorbent in diapers is made of a fibrous phase (usually fluff pulp) and a cross-linked, three-dimensional superabsorbent polymer (SAP) network based on acrylic acid–acrylamide mixtures [5].

Our values of FSC for CNF with ~200 m^2^/g of specific surface of CNF are of the same magnitude as the values obtained by Brodin et al. for a foam [6]. In fact, this foam was produced with TEMPO-oxidized CNF with a similar NaClO amount (4.2 mmol NaClO/g). Similarly, they also observed the effect of the specific surface of the fibers (nanofibers, in this case) since they obtained lower FSC values as they decreased the amount of microfibrillated cellulose on the composite. In a similar context, CNF also exhibited better performance than fluff pulp and the commercial absorbent, since in all cases the nanostructured cellulosic fibers exhibited higher CRC than the reference materials. It is interesting to note that the commercial diaper (only one brand was analyzed) exhibited lower water retention capacity. In fact, SAPs for diapers have limited swelling capacity in order to promote water penetration into the fluff structure of the absorbent.

**Figure 7:**
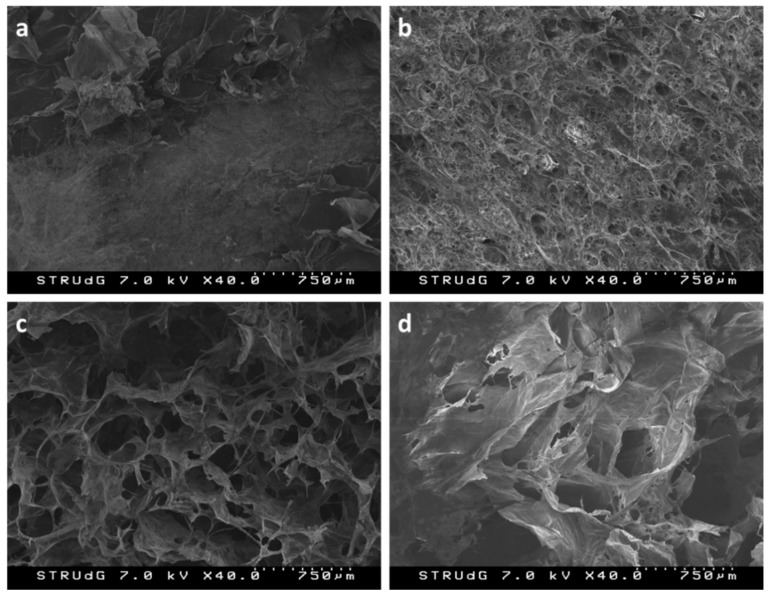
Field emission scanning electron microscopy (FE-SEM) images of aerogels prepared with CNF-5 (**a**), CNF-10 (**b**), CNF-15 (**c**) and CNF-25 (**d**).

The higher water absorption capacity of aerogels as the oxidizer amount was increased could also come from the differences on the structure and morphology of the developed aerogels. In principle, higher porosity of aerogels would lead to higher surface area thereof, allowing the penetration of the saline solution into the fibrous structure [28,50]. Although the most appropriate parameter to determine the effect of structure of aerogel on absorption properties would be the surface area thereof, the morphology of aerogels was investigated by means of FE-SEM (Figure 7). We found a high porosity in these structures that probably enabled the penetration of the saline solution into the aerogel and correlated with the high specific surface values of the prepared CNF and their values of FSC and CRC. In all cases, the typical honeycomb-like structure was found, as it has been reported in other works [58,59]. In fact, the structure of the CNF-5 aerogel exhibited a higher compaction of the CNF and, as the oxidation degree was increased, higher pore size was observed. These differences mainly come from the repulsive forces that CNF impart between them. The higher the oxidation degree is, the higher the carboxylate content and, thus, the higher the anionicity of the CNF.

However, as the oxidation degree increases, the surface area of CNF is also enhanced, allowing the creation of hydrogen bonding between them once water is removed. This might lead to less surface area of the aerogels, as it can be seen in Figure 7. The aerogels prepared from CNF-5 (Figure 7a), exhibited low porosity compared to those prepared with CNF at higher oxidation degrees, fact that may limit their capacity to retain water. On the opposite, those aerogels made of CNF-25 exhibited large pores, but CNF were less individualized, as it can be observed in Figure 7d. At moderate levels of NaClO during TEMPO-mediated oxidation, the resulting aerogels exhibited well distributed pores with increasing size, making apparent the difference between CNF-10 and CNF-15. Cervin et al. observed that the absorption capacity aerogels increased as the pore size was increased, since the penetration of the liquid (water/oil mixtures in this case) was easier and there were less steric effects [39]. In a similar context, Mendoza et al. observed that increasing the CNF consistency prior to aerogel preparation in one order of magnitude (from 0.3 to 3.0 wt%), had a significant effect on pore availability (about 50% decrease) and, thus, on the FSC of the aerogels [50].

There is no doubt that the oxidation degree, together with the cationic demand and the specific surface of CNF, directly affect the structure of the resulting aerogels. In addition, process parameters as well as solid content of the initial CNF gel have been reported to have a significant effect on the absorption properties [36,50,57].

**Figure 8:**
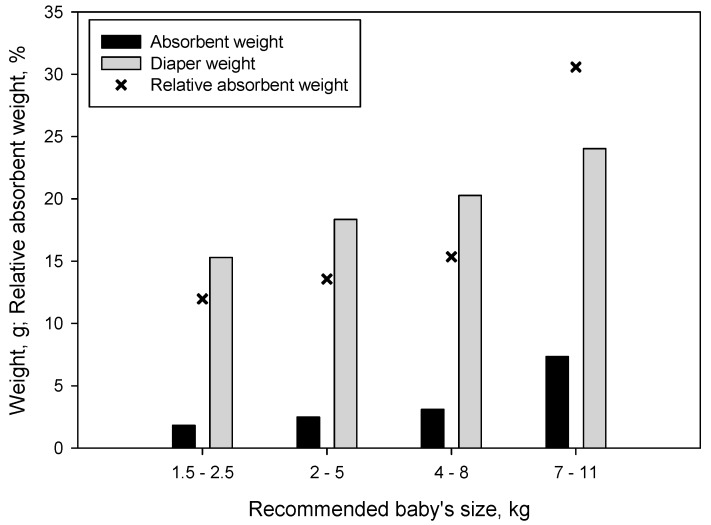
Diaper weight and amount of absorbent as function of the diaper size.

In order to further develop this work, four different diaper sizes were selected from one leading brand in Spain and Portugal. The amount of absorbent was quantified and compared to the total weight of the diaper (Figure 8). We found that a greater amount of absorbent was present as the size of the diaper increased. Surprisingly, the relative amount of absorbent, understood as the ratio between the amount of absorbent and the total weight of the diaper, also increased with the size of the diaper. The larger increase was for diapers recommended for babies ranging 7–11 kg.

Thus, assuming that the FSC is an indicator of the amount of urine that a diaper can retain when in use, and, knowing the amount of absorbent that the analyzed diapers contain, we generated a table that shows the amount of saline solution that each diaper can hold (Table 2). Taking into account this absorption capacity, together with the different FSC reported in Figure 6 (after 60 min of immersion), the amount of each type of CNF required for each diaper size was also calculated.

Table 2 shows that the higher the oxidation degree of CNF, the lower the amount of absorbent was required. In fact, the size 3 diapers deserve special attention since they exhibited the highest absorption capacity. While commercial diapers contain 7.35 g of absorbent (composite of fluff pulp and SAP), this amount could be decreased to 5.59 g if CNF-10 were used, and even lowered to 4.76 if CNF-25 were to be used. Hence, the cost of CNF production would represent approximately 0.04 €/diaper and 0.06 €/diaper for CNF-10 and CNF-25, respectively. However, establishing a comparison between production costs is still something difficult: on the one hand, the drying techniques of CNF are still inefficient and further research needs to be developed in this regard. On the other hand, data on the production costs of the commercial absorbents is difficult to obtain due to industrial secret and intellectual property protection. Nonetheless, it can be already stated that the use of TEMPO-oxidized CNF as superabsorbent for diapers can become a reality, especially if drying techniques are further developed. The average price of a diaper in Spain is 0.21€ (regardless of the size), meaning that in the case of size 3, the use of CNF-10 would represent a 17.14% of the total cost of the diaper and the use of CNF-25, a 26.67%. These costs are even lower for smaller diapers, but also in the case of the commercial diapers.

Finally, we determined the percentage of liquid (saline solution, 0.9% NaCl) release of the obtained aerogels under compressive stress at 50% and 100% deformation, as shown in Table 3.

The compression strength increased when higher amounts of NaClO were used, achieving a value of 3.06 kPa in the case of CNF-25. We believe that the higher number of inter-CNF bonds may promote the dimensional stability of the resulting aerogels, as well as the honeycomb-like structure thereof. Thus, the higher liquid retention capacity of the aerogels as the oxidation degree might come from two effects: on the one hand, the higher absorption capacity of the CNF, as observed in the WRV analyses. On the other, the higher dimensional stability due to the higher amount of inter-CNF bonds. In fact, when 100% deformation was applied, aerogels made of CNF-5 and CNF-10 disintegrated due to the compression stress. Again, the higher number of inter-CNF bonds prevented the release of liquid under stress, which was retained inside the three-dimensional structured network of cellulose nanofibers. Thus, this loss of water should be taken into account when considering the use of CNF as superabsorbent for diapers. Nonetheless, the results obtained for CNF (except in the case of CNF-5) are still higher than the FSC of the fluff pulp and the diaper absorbent even under stress, showcasing the feasibility of their use in such applications.

## 4. Conclusions

In this work we found that the higher the oxidation degree of CNF, the higher their specific surface was. This led to more stable structures with higher absorption capacity in the presence of water. In all cases, the obtained CNF exhibited higher FSC than a commercial fluff pulp (ranging from 117.62% to 245.21% higher) and higher than the diaper absorbent, except for CNF-5 (ranging from 31.56% to 54.55%). In addition, a similar effect was found when CRC was analyzed, since all the different CNF exhibited a better behavior than the diaper absorbent and the commercial fluff pulp. The production costs of CNF revealed that it is feasible to use such nanostructured superabsorbent for diaper production if appropriate drying techniques are developed. Finally, the percentage of liquid release under compressive stress was analyzed and resulted in a value with a range of 12.19% to 26.41% when 50% deformation was applied and CNF-25 and CNF-5 were used, respectively. When 100% deformation was applied, this value was 26.12% and 38.65% for CNF-25 and CNF-15, respectively. In both cases, the absorption capacity under stress was higher than the FSC of the commercial diaper when no stresses were applied, suggesting again the high potential of CNF for this application. Finally, further research must be conducted in order to understand the effect of the CNF characteristics on the aerogels structure and, at the same time, on absorption properties.

## Figures and Tables

**Table 1:** Characterization of CNF obtained with different oxidizer amounts (NaClO).

NaClO (mmol/g)	T at 600 nm (%)	Yield of Fibrillation (%)	DP (-)	WRV (gH_2_O/g)
5	80.2 ± 1.2	94.61 ± 1.63	488 ± 21	8.21 ± 0.31
10	81.8 ± 1.6	97.82 ± 2.16	232 ± 18	11.03 ± 0.53
15	84.7 ± 2.1	98.89 ± 1.05	199 ± 9	12.79 ± 0.44
25	88.0 ± 1.7	99.13 ± 0.68	169 ± 6	13.08 ± 0.47

T: transmittance; DP: degree of polymerization; WRV: water retention value.

**Table 2:** Absorption capacity of commercial diapers and the required amount of CNF at each oxidation degree.

Diaper Size	Recommended Baby’s Size (kg)	Absorption Capacity (g)	Required Amount of Absorbent (g)				
			Commercial	CNF-5	CNF-10	CNF-15	CNF-25
0	1.5–2.5	106.69	1.83	1.88	1.39	1.26	1.18
1	2–5	145.17	2.49	2.56	1.89	1.72	1.61
2	4–8	181.31	3.11	3.19	2.36	2.15	2.01
3	7–11	428.51	7.35	7.54	5.59	5.07	4.76

**Table 3:** Compression test at 50% and 100% deformation of the different aerogels.

NaClO (mmol/g)	50% Deformation		100% Deformation	
	σ_c_^A^ (Pa)	Liquid Release (%)	σ_c_^A^ (Pa)	Liquid Release (%)
5	590 ± 42	26.41 ± 1.26		*Disintegrated*
10	980 ± 63	23.73 ± 1.83		*Disintegrated*
15	1860 ± 101	19.65 ± 0.97		38.65 ± 2.10
25	3060 ± 176	12.19 ± 1.05		26.12 ± 1.91

σ_c_^A^: compression strength; σ_c_^A^ was not measured at 100% deformation to avoid the effect of the compression between plates.
